# National survey of the injury prevention activities of children's centres

**DOI:** 10.1111/hsc.12059

**Published:** 2013-07-09

**Authors:** Michael C Watson, Caroline A Mulvaney, Denise Kendrick, Jane Stewart, Carol Coupland, Mike Hayes, Persephone Wynn

**Affiliations:** 1 Faculty of Medicine and Health Sciences, School of Nursing, University of NottinghamNottingham, UK; 2 Division of Primary Care, Faculty of Medicine and Health Sciences, University of NottinghamNottingham, UK; 3 The Child Accident Prevention Trust (CAPT)London, UK

**Keywords:** children's centres, injuries, national survey

## Abstract

Children's centres were established across England to provide a range of services including early education, social care and health to pre-school children and their families. We surveyed children's centres to ascertain the activities they were undertaking to prevent unintentional injuries in the under fives. A postal questionnaire was sent to a sample of children's centre managers (*n* = 694). It included questions on current activities, knowledge and attitudes to injury prevention, health priorities and partnership working. Responses were received from 384 (56%) children's centres. Overall, 58% considered unintentional injury prevention to be one of the three main child health priorities for their centre. Over half the respondents (59%) did not know if there was an injury prevention group in their area, and 21% did not know if there was a home safety equipment scheme. Knowledge of how child injury deaths occur in the home was poor. Only 11% knew the major cause of injury deaths in children under five. Lack of both staff time and funding were seen as important barriers by children's centre staff to undertake injury prevention activities. Nearly all stated that training (97%) and assistance with planning injury prevention (94%) would be helpful to their centres. Children's centres need further support if they are to effectively tackle this important public health area.

## What is known about this topic

- Unintentional injury is the major public health challenge facing pre-school children in England today.
- Children's centres have important roles to play in relation to the prevention of unintentional injuries.

## What this paper adds

- This is the first study analysing the unintentional injury prevention activities of children's centres.
- There is a wide variation in the priority accorded to child unintentional injuries and the prevention activities undertaken by different children's centres.
- Children's centres may need support if they are to fulfil their child unintentional injury prevention roles.

## Introduction

Unintentional injury is the major public health challenge facing pre-school children in England today (Audit Commission/Health Care Commission 2007, World Health Organization 2008, Office for National Statistics 2011, Stone 2011) and is responsible for preventable deaths, disability and a great deal of suffering for children and their families (British Medical Association 2001, Department for Children Schools & Families 2009, Walter 2010). However, those who are charged with developing and implementing strategies to prevent unintentional injury at a local level often find it difficult to do so (Speller *et al*. 1995, Towner *et al*. 1998, Watson & White 2001, Kendrick *et al*. 2003, Audit Commission/Health Care Commission 2007, Baggott 2011). The 2007 Audit Commission/Healthcare Commission report ‘Better safe than sorry’, emphasised that there was no single, clear, cross-governmental statement, drawing together what needed to be done to reduce unintentional injury (Audit Commission/Health Care Commission 2007). At a local level in the National Health Service (NHS), the report found little evidence of a systematic approach to develop, implement and monitor programmes to prevent unintentional injuries in children.

Sure Start Children's Centres were an important part of the last UK Government's childcare strategy and were established across England between 2004 and 2010 to improve health and educational outcomes for children (Children Schools & Families Committee 2010). They are places where children and families can receive information, support and co-ordinated services from a range of professionals (Her Majesty's Treasury's 2004). The current government's Department for Education is clear that children's centres can play an important role in supporting the evidence-based Healthy Child Programme, and their statutory guidance contains a range of health topics including ‘advice on accident and injury prevention’ (Department for Education 2010). Children's centres thus have the potential to make significant contributions to home safety for children.

Despite numerous recent publications reporting the evolution and impact of children's centres (Avis *et al*. 2007, Hutchings *et al*. 2007, Melhuish *et al*. 2008, 2010, The National Evaluation of Sure Start Research Team 2008, MacNeill 2009, Children Schools & Families Committee 2010, Department for Education 2010, Baggott 2011), their role in injury prevention has received little attention in the literature. This study aims to describe and quantify the injury prevention activity being undertaken by children's centres across England.

## Methods

### Survey development

Relevant questions were generated by the study group of researchers; where possible, questions were taken from previous injury prevention surveys that were targeting other professional groups (Watson & White 2001, Kendrick *et al*. 2003, Watson *et al*. 2007). An assessment of face validity was carried out by the faculty members who had no training in injury prevention, and a check on content validity was undertaken by experts within the study group (Litwin 1995). The reliability of the questions was addressed by structuring questions so that they followed the principles of questionnaire design; using experts to advise; and careful piloting (Oppenheim 1992, Salant & Dillman 1994). A total of 10 children's centre managers from across the country who were not part of our final sample were selected to be part of the pilot and this resulted in minor modifications to the questionnaire.

The final questionnaire consisted of pre-coded closed questions and open-ended questions and covered the following areas:

- characteristics of children's centres;
- health priority areas;
- injury prevention activity;
- knowledge and attitudes towards injuries and their prevention;
- barriers and facilitators to injury prevention activity;
- partnership working.

### Sampling

The study population comprised managers of children's centres in England. Stratified cluster sampling was used to identify children's centres. Three primary care trusts (PCTs) were sampled at random from each Strategic Health Authority in England (*n* = 10) and all children's centres (*n* = 694) within the sampled PCTs were selected.

### Survey execution

The initial mailing took place in March 2010 and consisted of a postal questionnaire, a covering letter and a prepaid envelope. Three reminders were used to improve the response rate (McColl *et al*. 2001, Edwards *et al*. 2002).

### Data entry and analysis

Data were entered into a Microsoft Access database. A random 1 in 10 sample of questionnaires was double entered, and discrepancies with the original data were identified and corrected. The data error rate was 0.19%. Data were analysed using StataSE 11 (StataCorp., College Station, TX, USA). Binary and categorical variables were summarised using frequencies and proportions.

### Ethics

Approval for the study was granted by the North Nottinghamshire Research Ethics Committee (study reference number = 09/H0407/44).

## Results

### Response rate and respondents' characteristics

A total of 384 completed questionnaires were returned (55.8%). Of 694 questionnaires posted, five were returned undelivered and one was no longer a children's centre. A total of 39% were centres that were established in phase 1 (2004–2006), 54% were phase 2 (2006–2008) and 6% were phase 3 (2008–2010).

### Priority areas

Overall, 58% considered injury prevention to be one of the three main priority areas for children's health for their centre. Other topics listed in their top three priorities included ‘healthy diet/healthy lifestyle’ (81%), ‘breastfeeding’ (24%), ‘mental health/emotional well-being’ (11%), ‘child protection’ (11%), ‘dental health’ (11%), ‘ante/postnatal support’ (11%) and ‘smoking cessation support’ (8%).

### Strategies

While 47% of respondents stated that their centre had a written child injury prevention strategy, 43% did not and 10% of respondents did not know. In addition, 36% of respondents stated that their PCT/Local Authority had a written strategy, but the majority of respondents (61%) did not know.

### Knowledge and attitudes

Knowledge of the main cause of child injury deaths in the under fives in the home was poor, with only 11% (38 of 348) being correct (choking and suffocation). Similarly, only 33% (115 of 350) knew that falls were the major cause of non-fatal unintentional injuries to children under five in the home.

Respondents were asked for their attitudes towards injury prevention (Table 1). Nearly all respondents agreed that most child injuries are preventable (95%). Although 88% tended to see the responsibility of injury prevention lying with the parent/carer, the respondents also thought that there were important roles for children's centres. Nearly everyone (99%) was in agreement that children's centres can be effective in preventing injuries, and the majority thought that they should be involved in lobbying and campaigning (78%), and collecting data on injuries (87%).

**Table 1:** Attitudes towards injury prevention amongst respondents, *N* (%)

Statement	Strongly agree 1	Agree 2	Not sure 3	Disagree 4	Strongly disagree	Agreement 1 + 2	Disagreement 4 + 5
Injury prevention is predominantly the responsibility of the parent/carer [15]	115 (31.2)	208 (56.4)	2 (0.5)	31 (8.4)	13 (3.5)	323 (87.5)	44 (11.9)
Most child injuries are preventable [5]	110 (29.0)	248 (65.4)	4 (1.1)	16 (4.2)	1 (0.3)	358 (94.5)	17 (4.5)
Children's centres can be effective in preventing injuries [3]	136 (35.7)	241 (63.3)	1 (0.3)	3 (0.8)	0	377 (99.0)	3 (0.8)
Other agencies have a greater responsibility for injury prevention than children's centres [5]	15 (4.0)	74 (19.5)	46 (12.1)	223 (58.8)	21 (5.5)	89 (23.5)	244 (64.4)
National and regional agencies are better placed than local ones to educate the public about preventing injuries [19]	7 (1.9)	38 (10.4)	43 (11.8)	250 (68.5)	27 (7.4)	45 (12.3)	277 (75.9)
Children's centres should be involved in lobbying or campaigning on local safety issues [17]	76 (20.7)	209 (57.0)	3 (0.8)	42 (11.4)	37 (10.1)	285 (77.7)	40 (10.9)
It is important for our centre to collect data on injuries [6]	120 (31.8)	207 (54.8)	5 (4.8)	28 (7.4)	5 (1.3)	327 (86.5)	33 (8.7)

Values within brackets indicate missing values.

In terms of perceived effectiveness of injury prevention interventions for children aged under 5 years, both providing home safety equipment and one-to-one home safety advice by centre staff were considered either very effective or effective by most respondents (89%). A large number of respondents (86%) also considered group home safety advice from centre staff to be either very effective or effective at reducing injuries. A total of 69% of respondents deemed media campaigns on home safety to be a very effective or effective intervention. However, providing leaflets (without additional advice) was considered very effective or effective by only 40% of respondents.

### Activities

Overall, 97% stated that their centre was involved in some form of injury prevention. While nearly all displayed posters on child safety (97%) and took part in Child Safety Week (93%), fewer were involved in media work (17%), issuing first-aid kits (15%), or lobbying or campaigning on local safety issue(s) (34%). However, just over three quarters (76%) invited outside speakers to talk to parents, with the three most common topics covered being ‘home safety’, ‘road safety’ and ‘fire safety/burns and scalds’.

In relation to fire and burns and scalds prevention, the children's centres provided advice on a range of topics in various forms (Table 2). The main topic presented as one-to-one advice was smoking cessation (66%), while advice on handling hot drinks was most often imparted to groups (65%) and general fire prevention advice was usually presented using leaflets (79%).

**Table 2:** Advice provided by children's centres on fire safety and burns and scalds

Topic	One-to-one advice *N* (%)	Advice in groups *N* (%)	Leaflets *N* (%)	No advice *N* (%)	Don't know *N* (%)
General fire prevention	117 (30.5)	175 (45.6)	303 (78.9)	17 (4.4)	4 (1.0)
Handling hot drinks	163 (42.5)	248 (64.6)	243 (63.3)	16 (4.2)	4 (1.0)
Using cigarettes, lighters and matches	125 (32.6)	128 (33.3)	182 (47.4)	72 (18.8)	26 (6.8)
Bonfire and firework safety	66 (17.2)	160 (41.7)	252 (65.6)	44 (11.5)	13 (3.4)
Barbecue safety	38 (9.9)	75 (19.5)	102 (26.6)	161 (41.9)	34 (8.9)
Cooking safety	151 (39.3)	217 (56.5)	193 (50.3)	43 (11.2)	8 (2.1)
Using candles safely	64 (16.7)	90 (23.4)	98 (25.5)	140 (36.5)	39 (10.2)
Electrical safety	98 (25.5)	138 (35.9)	192 (50.0)	62 (16.2)	23 (6.0)
Handling hot irons safely	90 (23.4)	99 (25.8)	126 (32.8)	100 (26.0)	38 (9.9)
How to make a fire escape plan	87 (22.7)	129 (33.6)	144 (37.5)	87 (22.7)	35 (9.1)
Smoking cessation	255 (66.4)	219 (57.0)	300 (78.1)	7 (1.8)	5 (1.3)

Centre managers were asked to indicate which fire prevention, and burns and scalds activities were carried out by the children's centres. The most common activity was the provision of fireguards (33%) with fewer centres performing fire home safety risk assessments (23%) and providing (17%) and fitting (10%) smoke alarms. Only nine centre managers reported that they provided fire extinguishers or fire blankets. Three centres (1%) provided an exchange service of chip pans for deep fat fryers and only 1 (0.3%) provided an electric blanket checking/exchange service.

In response to the question ‘Is there a home safety equipment scheme in your area?’, nearly two-thirds (64%) answered yes, but approximately one-fifth (21%) did not know if there was such a scheme. Of those that had a scheme, 61% were part of the ‘Safe At Home’ national scheme organised by the Royal Society for the Prevention of Accident. Half (50%) the schemes had been in operation less than 18 months at the time of the survey. However, 14% had been in operation for more than four and a half years.

Over half the schemes (58%) operated from the children's centre. The types of equipment covered by schemes included stairgates (91%), fireguards (71%), cupboard locks (55%), window catches (47%) and corner cushions for tables (42%) The majority of the schemes provided free (69%) or low-cost equipment (18%). A smaller number loaned equipment (13%). Most schemes (84%) delivered equipment to homes and fitted equipment (78%).

### Joint working

Only 15% of respondents knew of an organised group specifically for child injury prevention in their area; the majority (59%) stated that they did not know. However, respondents stated that they were working with a range of organisations on injury prevention, including Community Nursing Services (86%), Fire and Rescue Service (69%), Road Safety (61%), Local Authorities (54%) and voluntary organisations (32%). Only 10% of centres worked with staff at Accident and Emergency departments.

From the responses of children's centre managers, it is clear that staff frequently refer families to other agencies for advice and support in fire, and burns and scalds prevention. Almost all centres refer families to NHS smoking cessation services (96%). Many centres refer families to the Fire and Rescue Services for advice and support on smoke alarms (86%) and for fire home safety risk assessments (85%). Approximately, a quarter of centres refer families to the Fire and Rescue Services for fire extinguishers/fire blankets (26%), for an electric blanket checking/exchange service (25%) and for exchange of chip pans for deep fat fryers (25%).

### Barriers and enabling factors to injury prevention work

The main barriers and enabling factors to injury prevention activities by the children's centre staff are shown in Table 3. Lack of capacity in terms of staff time (34%) and lack of funding (29%) were the two most frequently mentioned barriers. In terms of enabling factors, the two most frequently mentioned factors were access to families (29%) and working with other agencies (29%).

**Table 3:** Barriers and enabling factors to injury prevention work in the children's centres

	*N* (%)
Barriers	
Lack of capacity/lack of staffing	131 (34.1)
Lack of funding	111 (28.9)
Difficult to access certain families	75 (19.5)
Lack of staff training/lack of staff knowledge	59 (15.4)
Lack of data	57 (14.8)
Lack of multi-agency working/lack of information sharing between agencies	49 (12.8)
Lack of space to store equipment/display leaflets	22 (5.7)
Language problems/poor literacy	17 (4.4)
Enabling factors	
Access to families/accessible to families/good relationships with families	113 (29.4)
Working with other agencies	110 (28.7)
Availability of free/low-cost home safety equipment	51 (13.3)
Trained, knowledgeable staff	48 (12.5)
Availability of leaflets to distribute	33 (8.6)
Dedication/commitment of staff	31 (8.1)

### Future support

In relation to potential future support, nearly all stated that training (97%), provision of educational materials (95%), examples of good practice (94%) and assistance with planning injury prevention (94%) would be of use to their children's centre. Managers also reported that support for working with partners (89%) and communities (88%) would be helpful. Many (85%) also felt that help with evaluation would be useful to their centre.

## Discussion

### Main findings of this study

This is the first national study to focus on the unintentional injury prevention activities of children's centres, and we found variation in the priority accorded to child unintentional injuries and the prevention activities undertaken. Although managers had positive attitudes towards potential injury prevention roles, they had gaps in knowledge about both injury prevention and important local initiatives. Despite recent guidance, child injury prevention appears to be a neglected area within children's centres compared to the scale of the problem.

### What is already known on this topic

There are no published studies on the unintentional injury prevention activities of children's centres with which to compare our work. However, the findings are in agreement with two earlier surveys that investigated the injury-prevention activities of health authorities (Watson & White 2001) and primary care trusts (Kendrick *et al*. 2003). All three studies have raised the issue of capacity, the need for good data and the priority accorded to injury prevention work.

In relation to the importance of collecting data on injuries, the health authority survey found lack of data/quality of data to be the third most important barrier to injury prevention work (Watson & White 2001). In agreement with this, 74% of primary care group survey respondents thought that it was important to collect data on injuries (Kendrick *et al*. 2003) and this finding is comparable to our finding of 78% of children's centre managers. The survey of health authorities found that the two main barriers to injury prevention work were lack of resources (60%) and low priority (52%) (Watson & White 2001), and the later survey of PCTs concluded that:

…it seems accidental injuries are, at present, a neglected area…. (Kendrick *et al*. 2003, p. 388)

Similarly, our survey found the two main barriers to be lack of capacity (34%) and lack of funding (29%).

There has recently been a number of national publications from different organisations that have indicated the priority that should be given to child injury prevention and over half the respondents in this survey appeared to be in agreement with them (Audit Commission/Health Care Commission 2007, Department for Children Schools & Families 2009, National Institute for Health & Clinical Excellence 2010a). However, over 40% of the respondents did not have child injury prevention as one of their top three priorities, and the activities and knowledge of some respondents indicate that they may not be giving this public health area the attention it requires. It should be noted that the recent National Institute for Health and Clinical Excellence documents provide clear evidence-based recommendations about who should take action and the actions they should take (National Institute for Health & Clinical Excellence 2010a,2010b, 2013).

In relation to collaboration with other key groups in the field, the children's centres appear to be working with individual organisations rather than being part of multi-agency partnerships. Such partnerships have been recommended by public health specialists and injury prevention experts for many decades (World Health Organization 1978, 1986, Department of Health 1993a, Child Accident Prevention Trust 2003); details of such partnerships are also included in recent guidance (Audit Commission/Health Care Commission 2007, Department for Children Schools & Families 2009, National Institute for Health & Clinical Excellence 2010a). Effective partnership work is a complex process and requires a wide range of skills and a great deal of commitment (Department of Health 1993b, Watson 1994, Scriven 1998). Children's centres are likely to need help in developing and maintaining effective multi-agency partnership work.

It is of concern that the majority of managers were unaware of injury prevention activities such as organised injury prevention partnerships in their local area. In addition, many were unaware of the main causes of mortality and injury to children in their homes. However, it is positive that nearly all thought that child injuries are preventable and that children's centres can be effective in this area. Moreover, many stated that they would like assistance to improve their work in this area.

Over the years, guidance has consistently recommended the need for local injury prevention co-ordinator posts to raise awareness and facilitate systematic strategic approaches to injury prevention (Hogg 1996, Towner *et al*. 1998, Audit Commission/Health Care Commission 2007, Department for Children Schools & Families 2009, National Institute for Health & Clinical Excellence 2010b). Co-ordinators could work with staff in children's centres to ensure that they have the right level of knowledge, skills and awareness about specific injury prevention activities and partnerships in their area.

### Limitations of this study

Although the response to this survey is similar to that of surveys of other occupational professional groups (Cook *et al*. 2009), a response rate of 56% raises the possibility of non-response bias. That is, those responding may have been more interested in injury prevention and also may have been more likely to undertake injury prevention activities. If this is the case, the findings may overestimate the injury prevention activity being undertaken by children's centres, but this would not alter the conclusions. However, it is also conceivable that managers concerned about what they perceive as a lack of commitment to injury prevention in their centres might be more inclined rather than less inclined to respond. Therefore, the net effect of these potentially contradictory biases is unpredictable.

It is important to note that the information gathered was self-reported and although no check was made with a ‘criterion of truth’ (Belson 1986), care was taken in the design of the questionnaire, including using questions, where possible, that had been published in articles in peer-reviewed journals, having a pilot study, and utilising the expertise within the team to critique the data collection tool in terms of relevance and validity. This resulted in a questionnaire that was a clear and simple tool to collect information.

## Conclusion

This is the first study analysing the unintentional injury prevention activities of children's centres and the knowledge and attitudes of their managers. The quantitative nature of this study has enabled us to gain a broad picture, rather than an in-depth one. Nevertheless, this study provides useful insights into the injury prevention activities of children's centres in England. The managers thought that there were important roles for children's centres in injury prevention and that most child injuries are preventable. However, many managers had gaps in knowledge about injuries and local prevention initiatives. Children's centres will need further support including training and resources, if they are to fulfil their potential in tackling this important public health issue.
